# Counterfactual thinking in patients with amnesia

**DOI:** 10.1002/hipo.22323

**Published:** 2014-07-11

**Authors:** Sinéad L Mullally, Eleanor A Maguire

**Affiliations:** 1 Institute of Neuroscience, Henry Wellcome Building for Neuroecology, Newcastle UniversityNewcastle upon Tyne, United Kingdom; 2 Wellcome Trust Centre for Neuroimaging, Institute of Neurology, University College LondonLondon, United Kingdom

**Keywords:** counterfactual thinking, episodic memory, imagination, scene construction, amnesia, hippocampus

## Abstract

We often engage in counterfactual (CF) thinking, which involves reflecting on “what might have been.” Creating alternative versions of reality seems to have parallels with recollecting the past and imagining the future in requiring the simulation of internally generated models of complex events. Given that episodic memory and imagining the future are impaired in patients with hippocampal damage and amnesia, we wondered whether successful CF thinking also depends upon the integrity of the hippocampus. Here using two nonepisodic CF thinking tasks, we found that patients with bilateral hippocampal damage and amnesia performed comparably with matched controls. They could deconstruct reality, add in and recombine elements, change relations between temporal sequences of events, enabling them to determine plausible alternatives of complex episodes. A difference between the patients and control participants was evident, however, in the patients' subtle avoidance of CF simulations that required the construction of an internal spatial representation. Overall, our findings suggest that mental simulation in the form of nonepisodic CF thinking does not seem to depend upon the hippocampus unless there is the added requirement for construction of a coherent spatial scene within which to play out scenarios. © 2014 The Authors. Hippocampus Published by Wiley Periodicals, Inc.

## INTRODUCTION

Ruminating on alternative versions of reality—“what if I had become a musician instead of a scientist”—is known as counterfactual (CF) thinking (Kahneman and Tversky, 1982; Byrne, 2005). CF thinking is thought to play an important role in everyday cognition by informing and regulating our future behavior (Roese and Olson, 1995; Roese, 1997). Interestingly, CF thinking, like recalling the past and imagining the future, requires a person to simulate internal models of events and to compare these internally generated models with external reality (Knight and Grabowecky, 1995). A tight neural coupling between recollecting the past and simulating future or fictitious events has been observed (Schacter et al., 2012; Klein, 2013; Maguire and Mullally, 2013). In particular, the hippocampus, once synonymous with memory alone (Scoville and Milner, 1957), is now believed to play a key role in both cognitive functions. This raises the interesting question of whether the hippocampus is also required for CF thinking. Here an important distinction must be made between episodic CF thinking, which involves an individual's personal experiences, and nonepisodic or nonpersonal CF thinking which is not directly self-relevant (for a review see Schacter et al., in press). Hippocampal activation in healthy subjects during episodic CF thinking has been noted in several functional MRI studies (De Brigard et al., 2013; Van Hoeck et al., 2013). However, these studies could not address the question of whether the hippocampus is necessary for successful episodic simulation of alternative versions of reality, nor did they examine nonpersonal CF thinking.

Neither type of CF thinking has been investigated in patients with focal bilateral hippocampal damage and amnesia. Such patients are reported to have impaired episodic memory and episodic future thinking, whereas their ability to imagine nonpersonal past and future scenarios seems to be preserved (Klein et al., 2002; Andelman et al., 2010). Moreover, Rosenbaum et al. (2007) have shown that amnesic patients are able to simulate other people's mental states. Of note also is a selective impairment in the generation of self-relevant CF thoughts while CF use (in a nonpersonal task) was intact in patients with prefrontal lesions (Gomez Beldarrain et al., 2005). From this, it might be predicted that episodic but not nonepisodic CF thinking would be impaired in amnesic patients. However, other studies have found that both episodic (Race et al., 2011) and detailed nonpersonal prospection (Race et al., 2013) are impaired in patients with medial temporal lobe damage. These latter results, coupled with the inability of amnesic patients to mentally simulate any kind of fictitious spatially coherent scene (e.g., Hassabis et al., 2007; Mullally et al., 2012), leads to the alternative hypothesis that even nonepisodic CF thinking may be impaired in patients with bilateral hippocampal damage.

Both of these hypotheses propose that episodic CF thinking would be impaired in amnesic patients. The status of nonepisodic CF thinking is what differentiates the two predictions. Elucidating the status of CF thinking, and nonepisodic CF thinking in particular, in amnesic patients could provide important insights into the hippocampal mechanisms purported to underpin simulation, with further implications for understanding recollection of the past and imagination of the future. For instance, if nonepisodic CF thinking is impaired, then it would appear that the hippocampus facilitates a process common to CF thinking, prospection and memory such as the novel recombination of event details (Schacter et al., 2012), mental time travel (Buckner and Carroll, 2007), or the ability to mentally construct and replay, or pre-play, spatially coherent scenarios (Maguire and Mullally, 2013). If nonepisodic CF thinking is fully intact, despite appearing to involve processes purported to be hippocampal-dependent and heavily implicated in episodic memory and future-thinking (such as the simulation of internal models of events), then this could suggest that these processes are not in fact hippocampal-dependent.

In order to explore this further, we tested six patients (3 females; mean age 42 yr, range 32–63 yr; mean Full Scale IQ 106, range 99–112) with bilateral hippocampal damage and 10 matched control participants (4 females; mean age 42.9 yr, range 32–63 yr; mean Full Scale IQ 110, range 104–115). One additional patient (patient E from Mullally et al., 2012) could not participate in the full study but performed the second task (the CF Inference Task—detailed below) along with two additional matched control subjects. Each participant gave informed written consent to participation in accordance with the local research ethics committee. This cohort of patients and controls has been reported in detail elsewhere (see Mullally et al., 2012). In brief, neuropsychological assessment revealed dense anterograde and retrograde amnesia in the context of otherwise preserved cognitive function in the patients. Using two different methods—automated voxel-based morphometry (Ashburner and Friston, 2005) and manual segmentation—the site of the patients' damage appeared to be restricted to the hippocampus (mean bilateral hippocampal volume reduction of 32%) and did not extend into adjacent regions or anywhere else in the brain.

Ideally, we would have assessed both episodic and nonepisodic CF thinking in this study. However, our patients were profoundly amnesic for their past experiences. They were therefore unable to recall specific episodes involving themselves which they could then be asked to change in an episodic CF thinking paradigm. As such, we elected to focus solely on nonepisodic CF thinking. Indeed, given that the most uncertainty exists about the status of nonepisodic CF thinking in these patients, as outlined above, providing the first evidence of whether or not they can engage in this form of CF thinking is of substantial interest.

To assess nonepisodic CF thinking, we first devised a novel narrative-based paradigm—the CF Generation Task—which provided an assessment of CF thinking, CF simulation and causal reasoning. Second, we administered the CF Inference Task (Hooker et al., 2000), which has previously revealed impaired CF thinking in patients with schizophrenia (Hooker et al., 2000) and Parkinson's disease (McNamara et al., 2003), but notably not in patients with prefrontal cortex lesions (Gomez Beldarrain et al., 2005). Both tasks capitalize on the fact that CF thoughts are not only pervasive (Summerville and Roese, 2008) but also predictable. For instance, people usually alter exceptional as opposed to routine events that happened before an unexpected occurrence (Kahneman and Tversky, 1982; Gavanski and Wells, 1989), and preferentially focus on action-based rather than inaction-based antecedents (Kahneman and Tversky, 1982). Similarly, there is a tendency to mutate those aspects of an event where people believe they had control (Girotto et al., 1991), while in a temporal sequence of events the focus is typically on the first (Wells et al., 1987) and/or the most recent event (Tetlock and Parker, 2005).

**Fig 1:**
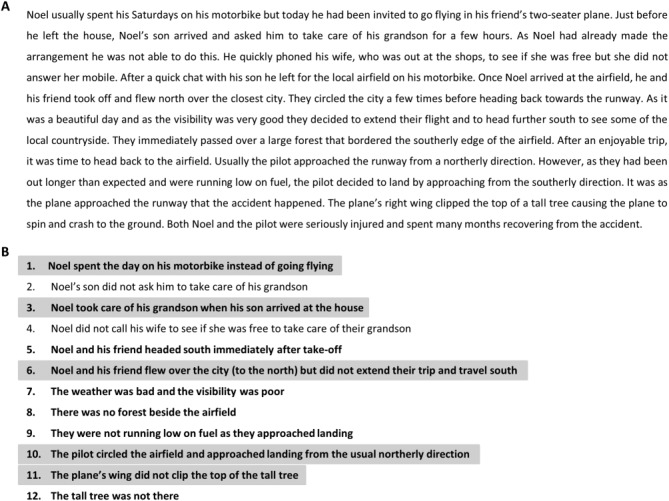
The CF Generation Task. A: The task narrative. B: The 12 altered antecedents. Ten of these altered antecedents could potentially change the outcome of the day's events, two could not (lure items: 2 and 4). Within the 10 causal antecedents, five represent mutation of the salient CF fault-lines (“causal + CF” items: 1, 3, 6, 10, 11, highlighted in gray for illustrative purposes only), whereas the other five items (5, 7, 8, 9, 12) were “causal-only” antecedents.

In the CF Generation Task, participants read a narrative about a fictional character Noel and his pilot friend who were involved in a plane crash (Fig. 1A). The sequence of events leading to the plane crash was structured so that it contained twelve seemingly salient “fault-lines” which could be readily altered. Within these 12 fault-lines, two would, if altered, not affect the outcome of the day's events (“lure” antecedents), five would, if altered, potentially change the outcome of the day's events but should not readily evoke CF thoughts (“causal-only” antecedents), while the remaining five fault-lines were both causal and CF in nature (they should readily evoke CF alternatives; “causal + CF” antecedents).

After reading the narrative, participants first recounted the story out loud. This ensured that all participants had processed the narrative in sufficient detail. Participants then identified the day's events that they believed Noel would have spent time thinking about while recovering from the accident, and that he would have wished turned out differently. All participants spontaneously identified the salient fault-lines within the story that they believed would have given rise to strong CF thoughts, and this did not differ between groups (patients 5.0, SD 2.1; controls 5.3, SD 1.95; *U* = 28.5, *Z* = −0.17, *P* = 0.87). Similarly, both groups selected the highly predictable fault-lines (“causal + CF” antecedents), while ignoring lure and “causal-only” antecedents. The apparently effortless manner in which the patients were able to identify salient CF fault-lines suggested that this form of thinking is unperturbed in hippocampal amnesia.

**Fig 2:**
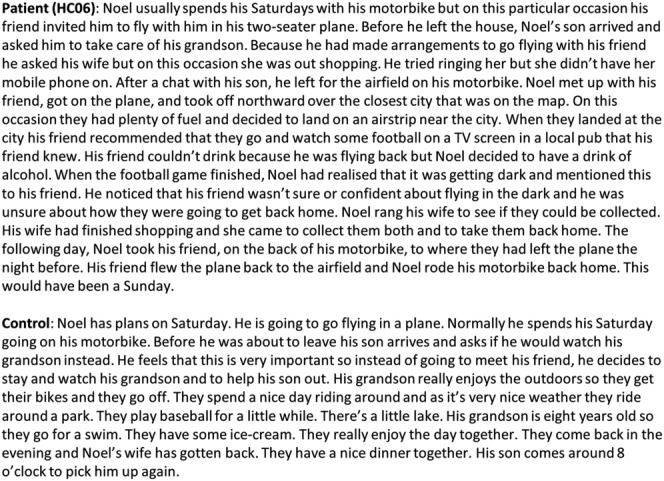
Examples of generated CF simulations. Representative excerpts from an example patient (top) and control participant (bottom).

**Fig 3:**
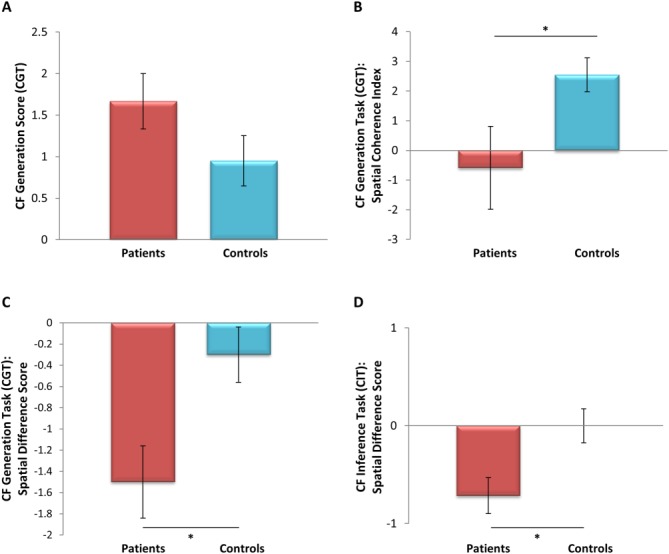
Indices from the CF Generation Task and the CF Inference Test. A: Both patients and controls found it easy to simulate CF alternatives in the CF Generation Task. B: By contrast, the patients rated the scenes that were evoked as they simulated CF alternatives as significantly more fragmented and less spatially coherent than controls. C: In addition, when explicitly instructed to select the altered antecedents from a pre-specified list (Fig. 1B), the patients selected more of the weakly spatial alternatives than strongly spatial alternatives. D: Similarly, in the CF Inference Test, the patients demonstrated a significant bias away from the selection of strongly spatial CF alternatives relative to the control group. * P<0.05. [Color figure can be viewed in the online issue, which is available at wileyonlinelibrary.com.]

In order to probe this further, we investigated whether patients could actually generate these alternate realities by asking participants to simulate two full CF alternatives of the day's events out loud. They therefore had to identify a specific fault-line, mutate it, and then simulate a coherent and logical sequence of events that could have arisen following this mutation. The simulations were scored in two ways. First, we asked if the participant generated a coherent narrative in which one specific event (CF fault-line) was mutated (Yes/No; for 100% of patients and 100% of controls the answer was Yes). Second, we asked whether the generated simulation demonstrated that the participant successfully recombined the previously given information with internally generated events/thoughts/actions to form a (1) coherent, (2) logical, and (3) novel sequence of events. A CF simulation was only considered acceptable if all three criteria were met. All the CF simulations provided by the patients and the controls met all three criteria, showing that both groups could easily simulate alternative scenarios. An example of a generated simulation from a patient and a control participant is shown in Figure 2. Difficulty was also formally assessed using a questionnaire that participants completed after simulating each CF alternative. From this, a CF generation score was derived whereby a negative score (range: −2.5 to +2.5) reflected an underlying difficulty generating spontaneous CF thoughts and simulating the corresponding CF alternative. Both groups scored comparably (*U* = 39.5, *Z* = −0.21, *P* = 0.83) and within the positive range (patients 1.67, SD 0.82; controls 0.95, SD 0.96; Fig. 3A). This suggests that the patients were not only able to identify predictable CF thoughts but they were also able to simulate with ease “what might have been.”

We then quantitatively assessed the nature of patients' CF thinking and compared this to their causal reasoning. In order to prevent confusion between the original narrative and the previously simulated CF alternatives, participants began this phase of the experiment by re-reading the original story and recounting it aloud. They were then presented with a list of the 12 altered antecedents (Fig. 1B) and asked to identify all of the altered antecedents from this list that could potentially have led to a different outcome for Noel. As previously mentioned, there were 10 causal fault-lines readily identifiable within the story narrative and two lure events. Both groups accurately identified the majority of these fault-lines (patients 9.0, SD 0.89; controls 9.5, SD 0.70; *U* = 20.0, *Z* = −1.18, *P* = 0.28) and correctly failed to select the lure items (patients 0.17, SD 0.41; controls 0.30, SD 0.67; *U* = 28.5, *Z* = −0.29, *P* =0.81).

Participants were then asked to identify the altered antecedents that Noel would have focused on in the days following the accident. All participants successfully identified a reduced number of antecedents (patients 4.5, SD 1.97; controls 5.7, SD 1.82; *U* = 22.0, *Z* = −0.89, *P* = 0.37), and selected the predicted “causal + CF” antecedents (Fig. 1B) more frequently than the “causal-only” antecedents (patients: “causal + CF” 4.0, SD 1.67; “causal-only” 0.5, SD 0.84; *Z* = −2.23, *P* ≤ 0.05; controls: “causal + CF” 4.1, SD 0.74; “causal-only” 1.6, SD 1.43; *Z* = −2.69, *P* ≤ 0.05). This preferential selection of the “causal + CF” antecedents occurred in spite of both groups having recognized, just moments earlier, the causal role of the other antecedents in the day's outcome, indicative of a sophisticated appreciation of CF processes in the patient group.

We then administered a second and separate CF Inference Test. This consisted of four vignettes (e.g., “Janet is attacked by a mugger only 10 feet from her house. Susan is attacked by a mugger a mile from her house”) that have been consistently found to evoke specific CF thoughts in healthy controls (Hooker et al., 2000). After reading each scenario, participants are presented with a question (e.g., “Who is more upset by the mugging?”) and selected a response from among three options (e.g., “Janet/Susan/Don't Know”). Both groups selected a comparable number of normative responses (patients 2.42, SD 1.39; controls 2.5, SD 1.62; *U* = 37.5, *Z* = −0.39, *P* = 0.69). Combined with the findings of the first CF Generation Task, this provides evidence that the complex cognition required for CF thinking in relation to nonpersonal episodes does not appear to depend upon the integrity of the hippocampus. Although it should be noted that performance for both groups is close to ceiling on the two measures of CF thinking, the fluidity and ease at which the patients engaged with these tasks strongly suggests that these processes are intact. However, when we probed deeper, a subtle difference between the patients and controls emerged.

In line with previous groups of patients (e.g., Hassabis et al., 2007), when tested by Mullally et al. (2012) our patients rated their attempts at imagining scenes as being spatially fragmented on a Spatial Coherence Index, which assesses the spatial coherence of imagined scenes. In this study, we used a modified version of the Spatial Coherence Index when participants explicitly simulated each CF scenario in the CF Generation Task. While control participants reported spontaneously imagining vividly unfolding scenes (2.97, SD 1.57), the patients rated their imagined scenes as significantly more fragmented and less spatially coherent (patients −0.056, SD 3.51; *U* = 12.0, *Z* = −1.96, *P* ≤ 0.05; Fig. 3B). Thus, the patients did not spontaneously evoke spatially coherent mental images of the unfolding narrative when generating their CF alternatives in the same way as the controls. We next asked whether this impacted upon their CF thinking.

We recruited two independent sets of healthy participants. The first set (*n* = 19; mean age 30.7 yr, SD 8.74; 6 females) read the CF Generation Task narrative and considered whether or not the 10 CF alternatives (Fig. 1B; lures excluded) could have altered the outcome. While doing this, they also reflected on whether they were visualizing the imagined scenarios. If 50% or more of the raters stated that they constructed a spatially coherent representation of the simulated scenario then that antecedent was considered to have a strongly spatial component. This yielded five strongly spatial (items 5, 6, 8, 10, 12) and five weakly spatial (items 1, 3, 7, 9, 11) antecedents. When we reanalyzed our patient and control groups' selection of altered antecedents using this classification, we found that although controls selected a comparable number of strongly spatial (2.70, SD 0.95) and weakly spatial (3.00, SD 1.05) antecedents (*Z* = −1.13, *P* = 0.26), patients selected significantly fewer strongly spatial (1.50, SD 0.84) than weakly spatial (3.0; SD 1.26) altered antecedents (*Z* = −2.26, *P* ≤ 0.05). This disparity between strongly spatial and weakly spatial altered antecedents was significantly different between the two groups (*U* = 8.5, *Z* = −2.46, *P* < 0.05; Fig. 3C). Moreover, when this analysis was restricted to the 5 “causal + CF” altered antecedents (two strongly spatial and three weakly spatial), this difference persisted (*U* = 15, *Z* = −2.02, *P* < 0.05). Thus, while the patients initially appeared to have performed in a comparable manner with controls, when their CF thinking was probed further, a subtle avoidance of strongly spatial simulations emerged.

A second set of raters (*n* = 9; mean age 33.4, SD 11.8; 3 females) considered whether in answering the question about each of the four CF Inference Test vignettes they pictured a scene. Two of the four vignettes were classified as strongly spatial (89% agreement) and two were classified as weakly spatial. Again, the patients selected significantly fewer normative responses for the strongly spatial vignettes (0.86, SD 0.69) than for the weakly spatial vignettes (1.57 SD 0.79; *Z* = −2.24, *P* = 0.025), a pattern that was not evident in the controls (strongly spatial: 1.25, SD 0.75; weakly spatial: 1.25, SD 0.97; *Z* = 0, *P* = 1). Once again this disparity between strongly spatial and weakly spatial altered antecedents was significantly different between the two groups (*U* = 17, *Z* = −2.35, *P* < 0.05; Fig. 3D).

In summary, here using two different tasks, we assessed nonepisodic CF thinking in patients with bilateral hippocampal damage and amnesia and found they were able to deconstruct reality, add in and recombine elements, change relations between temporal sequences of events, enabling them to determine plausible alternatives of complex episodes. Thus, these processes that enable nonepisodic CF thinking appeared to be intact, suggesting they are not dependent upon the hippocampus. It seems to us unlikely that the CF processes that are deployed for nonpersonal episodes and for first-person episodes would be different. However, our data do not allow us to rule out the possibility that the processes required for episodic CF thinking are hippocampal dependent. That being said, the normative, predictable changes that people make in nonepisodic CF tasks are also evident for personal past episodes (Kahneman and Tversky, 1982; Roese, 1997; Byrne, 2002), presumably because it is our first-hand experience of our own cognitive response to a similar situation that guides our nonepisodic CF inferences. It is therefore credible that patients with bilateral hippocampal damage, despite their dense episodic amnesia, could utilize episodically derived (pre-morbid) CF principles in everyday thinking and hence would, if they were able to recollect episodic events in sufficient detail, engage in episodic CF thinking. It is unclear to what extent core CF processes utilize semantic knowledge, scripts or other heuristics which may have originally been acquired in an episodic manner but which then become independent of the hippocampus (Klein, 2013; Mullally et al., 2014). Detailed probing and analysis of patients' outputs during CF thinking tasks may yield further important insights in this regard.

Our finding that the patients avoided CF simulations that required the construction of an internal spatial representation suggests that there may be at least one difference between the patients and controls in how they approached the tasks. Future work will be needed to corroborate these spatial findings, which are concordant with accounts that view space (O'Keefe and Nadel, 1978) or spatially coherent scenes (Maguire and Mullally, 2013) as fundamental to hippocampal functioning. Our finding that this hippocampal spatial contribution may pervade not just navigation or episodic memory/future thinking as has been suggested previously (Maguire and Mullally, 2013), but also even nonpersonal CF thinking, provides a further potential clue about how these disparate cognitive functions might be linked.

By necessity, we focused here on one form of CF thinking that involved nonpersonal episodes. However, there is much yet to learn about how the different types of CF thinking relate to each other and to the underlying brain anatomy. Overall, our results and those such as Rosenbaum et al. (2007), who showed that the ability to simulate or infer the intentions of others also does not require hippocampal integrity, reinforces the need to think more deeply about the nature of mental simulation and the circumstances under which the hippocampus becomes necessary.
